# Efficacy and safety of irinotecan combined with raltitrexed or irinotecan monotherapy for salvage chemotherapy of esophageal squamous cell cancer: A prospective, open label, randomized phase II study

**DOI:** 10.1002/cam4.6264

**Published:** 2023-06-16

**Authors:** Xichao Dai, Leilei Tao, Jinqiu Wang, Wenjuan Wu, Weigang Bian, Xichun Dai, Surong Chen

**Affiliations:** ^1^ Department of Oncology First people's Hospital of Yancheng Yancheng China; ^2^ Yancheng Clinical College of Xuzhou Medical University Xuzhou China; ^3^ The Fourth Affiliated Hospital of Nantong University Nantong China; ^4^ Yancheng Dafeng People's Hospital Yancheng China; ^5^ Department of Oncology Northern Jiangsu People's Hospital, Clinical Medical College of Yangzhou University Yangzhou China; ^6^ Department of Oncology Hongze People's Hospital of Huai'an City China

**Keywords:** esophageal squamous cell cancer, irinotecan, raltitrexed, salvage chemotherapy

## Abstract

**Background**: Esophageal squamous cell cancer (ESCC) accounts for approximately 90% of esophageal cancer cases in China. There are no standard regimens for second or third–line chemotherapy of metastatic squamous esophageal cancer. The objective of this study was to investigate the security and effectiveness of irinotecan combined with raltitrexed or irinotecan monotherapy for salvage chemotherapy of ESCC.

**Methods**: One hundred and twenty–eight patients with metastatic ESCC confirmed by histopathology were enrolled into this study. These patients had failure of the first–line chemotherapy combination of fluorouracil or platinum or paclitaxel and had not undergone chemotherapy with irinotecan or raltitrexed previously. Patients were randomly divided into irinotecan combined with raltitrexed group (experiment group) and irinotecan monotherapy group (control group). Overall survival (OS) and progression–free survival (PFS) were the primary endpoint.

**Results**: In the control group, the median PFS (mPFS) and median OS (mOS) of patients were 3.37 and 5.3 months. In the experiment group, mPFS and mOS were 3.91 and 7.0 months. There was statistical significance of PFS and OS between two groups (PFS *P* = 0.002, OS *P* = 0.01). In subgroup analysis, in the second–line treatment, the mPFS of control and experiment group, was 3.90 and 4.60 months, mOS was 6.95 and 8.5 months, which was statistically significant differences between the two groups. (PFS *P* = 0.001, OS *P* = 0.005), In the third–line and beyond treatment, mPFS of control and experiment group was 2.80 and 3.19 months, mOS were 4.5 and 4.8 months. But there was no significant difference of PFS or OS between the two groups (PFS *P* = 0.19, OS *P* = 0.31). There was no statistical significance of toxicity side effects between two groups.

**Conclusions**: The PFS and OS of irinotecan plus raltitrexed may be better than that of irinotecan monotherapy, especially in second line treatment, which should be confirmed with a phase III study including much more patients.

## 1 INTRODUCTION

The mortality rate of esophageal cancer ranks seventh among all kinds of cancer‐related deaths worldwide.^1^ Squamous cell carcinoma and adenocarcinoma are the principal histopathological types of esophageal cancer.^2^, ^3^ Surgery is still the best option for the resectable disease; however, 5 year survival rate ranges from 15% to 40% which is disappointing.^4^ Metastatic disease is present during diagnosis in about 60% of patients.^5^

As a result, the use of chemotherapy as the primary treatment has been implemented to enhance the chances of survival for patients with advanced or metastatic esophageal cancer. For advanced esophageal cancer, the primary chemotherapy treatments consist of combining fluorouracil or paclitaxel with platinum.^6^ However, the disease of most patients progress after experiencing 5–10 months remission. There are no standard regimens for second‐ or third‐line chemotherapy of metastatic squamous esophageal cancer. Currently, most of esophageal cancer clinical researches are based on adenocarcinoma at gastric or esophagogastric junction.^7^ However, esophageal squamous cell cancer (ESCC) accounts for approximately 90% of esophageal cancer cases in China.^8^ So, it urged us to find new regimens or therapeutic approaches of second‐line or beyond chemotherapy for ESCC.

For ESCC, as a stand‐alone treatment for esophageal carcinoma that is resistant to cisplatin and irinotecan, which inhibits topoisomerase I, has demonstrated a total response rate of 15%.^9^ More and more studies are exploring the new option of irinotecan combined with some chemotherapy drug in order to get better efficacy. Studies have shown that irinotecan upregulates expression of thymine phospholipase in tumor tissue, and irinotecan combined with fluorouracil drugs get synergistic effect.^10^

Raltitrexed, which is also known as ZD1694 or Tomudex, is a potent and enduring inhibitor of thymidylate synthase (TS). DNA synthesis and repair are highly dependent on the role of TS. Numerous clinical studies have implied an elevation in TS levels in cancerous tissues, which indicates that it plays a potential role in cancer development and advancement. Raltitrexed, a TS inhibitor, which sometimes is a substitute for 5‐Fu, combined with irinotecan is widely recognized that is an effective treatment for colorectal cancer.^11^ At the same time, raltitrexed was found to exhibit antitumor properties in various cancer types through its direct antiproliferation and induction of apoptosis effects in preclinical studies. These cancer types include malignant mesothelioma, head and neck cancer, liver cancer, cervical cancer, and stomach cancer.^12^, ^13^, ^14^, ^15^, ^16^, ^17^

Sun et al. carried out a prospective study and found that raltitrexed increases radiation sensitivity of ESCC.^18^ However, the effect of irinotecan combined with raltitrexed in ESCC still unknown. The aim of this study was to investigate the effectiveness and safety of using irinotecan alone or in combination with raltitrexed as rescue chemotherapy for ESCC through a prospective design.

## 2 METHODS AND POPULATION

**FIGURE. 1:**
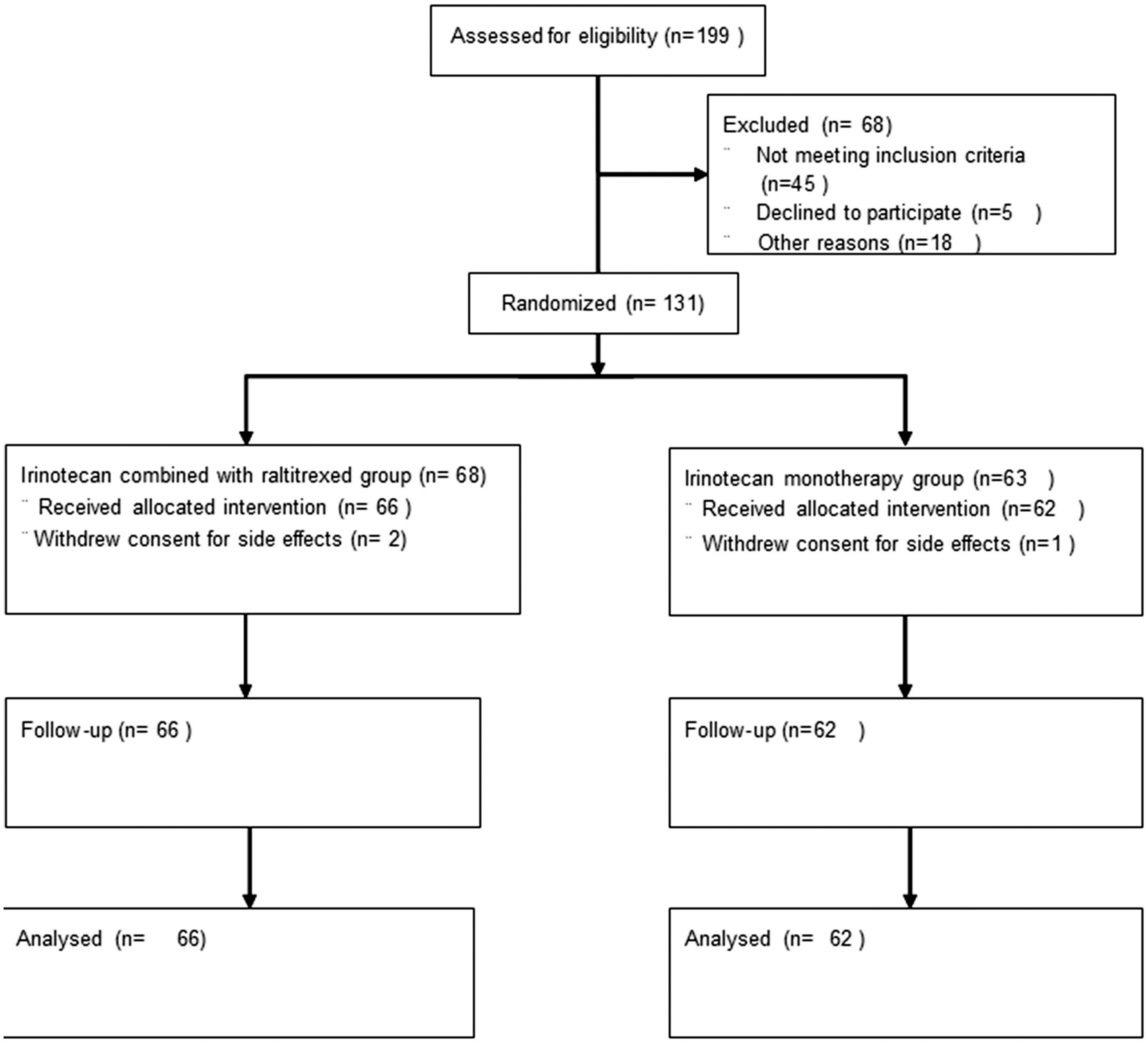
Flow diagram.

The research process is shown in Figure 1.

### 2.1 Study population

The phase II study was performed in two institutions (Northern Jiangsu People's Hospital and The First people's Hospital of Yancheng, China) between January 2016 and December 2020, registered in http://www.chictr.org.cn (identifier: ChiCTR1900027159). One hundred and twenty‐eight patients with unresectable or metastatic ESCC confirmed by histopathology were enrolled into this study. Patients were randomly divided into irinotecan combined with raltitrexed group (experiment group) and irinotecan monotherapy group (control group). The Institutional Review Board (IRB2020K016) of the First People's Hospital of Yancheng and the (KY2020001) Institutional Review Board of the Northern Jiangsu People's Hospital both approved the study. All the participants signed an informed consent before participating in the study.

To be eligible for the study, patients must meet the following criteria: (i) confirmation of ESCC diagnosis by biopsy; (ii) between 18 and 75 years of age; (iii) a performance status of ≤2 as determined by the Eastern Cooperative Oncology Group; (iv) the presence of at least one measurable lesion determined by the Response Evaluation Criteria in Solid Tumors (RECIST) 1.1 criteria; (v) failure of the first‐line chemotherapy combination of fluorouracil or platinum or paclitaxel; (vi) The patients had not undergone chemotherapy with irinotecan or raltitrexed previously; (vii) The patients had sufficient hematologic, renal, and hepatic function, as evidenced by an absolute neutrophil count greater than 1.5 × 109/l, a platelet count greater than 80 × 109/l, a total bilirubin level less than or equal to 1.5 × upper limit of normal, aspartate transaminase and alanine transaminase levels less than or equal to 2 × upper limit of normal, and a creatinine level less than or equal to 1.5 mg/dL. The criteria for exclusion: (i) no pathological diagnosis; (ii) previous chemotherapy with irinotecan or raltitrexed; (iii) cerebral or meningeal metastases.

### 2.2 Treatment

Before the first chemotherapy, several tests including physical examination, full blood count, clotting screen, urea and electrolytes, creatinine clearance, liver function tests, ECG, and chest CT scan were carried out for evaluating the disease. At the beginning of each treatment cycle, a complete blood count, liver function tests, and a chemistry panel were retested. Radiographic studies were repeated after every 2 cycles of treatment.

Irinotecan (Jiangsu Hengrui MedicineCo. Ltd.) was administered at 100 mg/m^2^ with intravenous (IV) injection over 30–90 min on day 1,8 or 180 mg/m^2^ on day 1. Every 21 days was a cycle of treatment, and raltitrexed (Jiangsu Zhengda Tianqing Pharmaceutical Co. Ltd.) was administered at 3 mg/m^2^ on day 1 in the same 21‐day schedule. The treatment cycle for chemotherapy was every 3 weeks and limited to a maximum of six rounds. In order to avoid the cholinergic syndrome linked to irinotecan, atropine 0.25 mg was administered subcutaneously before treatment. Patients were sent home with loperamide prescription to help with delayed diarrhea if necessary.

Patients were given a minimum of one therapy cycle until evident disease progression or severe toxicity occurred. The grading of all toxicity was in alignment with the National Cancer Institute Common Toxicity Criteria (CTCAE) 4.0. Dose adjustments were based on maximum toxicity observed from the prior cycle as previously described.^7^ If patients experienced grade 3 or 4 non‐hematological and hematological toxicity, the doses of both drugs were reduced 25%. Once a dose reduction occurred, doses were not re‐escalated. Patients were discontinued from treatment if they met any of the following criteria: pregnancy, noncompliance, unresolved grade 3 or 4 toxicity, or patient decision to discontinue treatment.

### 2.3 Observation indicators

Tumor was assessed with CT scan according to RECIST criteria^19^ within 7 days before starting treatment. Assessments of the same target lesion were then repeated using the same imaging technique every 6 weeks until the onset of progressive disease. To obtain a complete response (CR), all evaluable lesions must completely resolve. A partial response (PR) is when the sum of the longest diameter of target lesions decreases by 30% or more. In addition, progressive disease (PD) is indicated by an increase in the sum of the longest diameter of all target lesions by 20% or more. Neither PD nor PR was defined as a stable disease (SD). The objective response rate (ORR) = CR + PR. The disease control rate (DCR) = CR + PR + SD. A baseline CT scan was taken and subsequently, every two chemotherapy cycles, another scan was performed for the purpose of evaluating the response of the tumor.

OS and PFS were the primary endpoint. All patients were followed up once 6 weeks. OS was referred to the time from the start of treatment until death from any reasons or the last follow‐up visit, and the PFS was defined as the time from the start of treatment until disease progression/recurrence. The safety was the secondary endpoint for this study, the side effects of the two groups were compared.

### 2.4 Statistical analysis

The SPSS 22.0 software was utilized for performing statistical analyses, while a *p*‐Value < 0.05 was considered statistically significant. A power analysis by PASS 11.0 showed that the power of 62 specimens in control group and 66 specimens in the experiment group for clinical efficacy achieves 100% at a significance level of 5%, the power of 62 specimens in control group and 66 specimens in the experiment group for side effects achieves 100% at a significance level of 5%. The effectiveness of treatment and any adverse reactions were evaluated through either the Student's *t*‐test or chi‐square test. The curves for OS and PFS were calculated via the Kaplan–Meier approach, and the log‐rank test was used to compare the two curves.

## 3 RESULTS

### 3.1 Clinical baseline characteristics

From January 2016 to December 2020, a total of 128 patients from two institutions in China were enrolled into this study, 32 females and 96 males. Median age was 57.2 years (range 25–73 years). There were 54 cases of distant metastasis of one or two organs and 74 cases of more than two organs, the metastatic organs mainly included the liver, lungs, bones, and lymph nodes, other less common sites such as the spleen and adrenal glands, and intergroup differences were not statistically significant (*p* > 0.05). Among them, 66 cases received the second‐line therapy, 62 cases received the third‐line and beyond therapy. Previously used first‐line chemotherapy regimens mainly included fluorouracil‐based combination regimens and paclitaxel + platinum, with fluorouracil drugs including 5‐Fu, tegafur gimeracil oteracil potassium, capecitabine, etc., and platinum‐based drugs including cisplatin and nedaplatin. The specific combined treatment plans mainly included fluorouracil + paclitaxel, fluorouracil + cisplatin, fluorouracil + nedplatinum, paclitaxel + cisplatin, paclitaxel + nedaplatin. Among them, 32 cases had performance status (PS) = 0, 59 cases had PS = 1 and 37 cases had PS = 2 (Table 1).

**TABLE 1:** Clinical baseline characteristics of patients.

	Control	Experiment	*p* Value
Gender			
Male	45	51	0.55
Female	17	15	
Prior chemotherapy regimen			
Fluorouracil combined	17	18	0.88
Fluorouracil + cisplatin	12	13	
Fluorouracil + nedplatin	3	2	
Fluorouracil + paclitaxel	2	3	
Paclitaxel + platinum	15	16	
Paclitaxel + cisplatin	13	14	
Paclitaxel + nedplatin	2	2	
Both regimens were used	30	32	
Metastatic sites			
>2	38	36	0.44
≤2	24	30	
Site of metastatic disease			
Lung	28	30	1.00
Liver	14	15	
Bone	8	9	
Lymph nodes	51	55	
Other	11	13	
Age			
≤60 (25‐60)	30	34	0.72
>60 (61‐73)	32	32	
Line of Chemotherapy			
Second‐line	32	34	0.72
Third‐line and beyond	30	32	
PS			
0	15	17	1.00
1	29	30	
2	18	19	

### 3.2 Clinical efficacy

A total of 386 cycles were administered to 128 patients, with a median of 3 cycles per patient (range 2–6). All patients had completed evaluation for efficacy. No one achieved CR. In the control group, 16 patients had PR, 16 patients had SD and 30 patients showed PD, the ORR was 25.81%, and the DCR was 51.60%. In the experiment group, 18 patients had PR, 24 patients had SD, and 24 patients showed PD, the ORR was 27.27%, and the DCR was 63.64%. The two groups showed no significant differences (*p* > 0.05) (Table 2).

**TABLE 2:** Clinical efficacy of two groups (*n*, %).

		CR	PR	SD	PD	ORR	DCR
Experiment *n* = 66	Second‐line chemotherapy (*n* = 34)	0 (0.00)	11 (32.35)	16 (47.06)	7 (20.59)	11 (32.35)	27 (79.41)
	Third‐line and beyond chemotherapy (*n* = 32)	0 (0.00)	7 (21.88)	8 (25.00)	17 (53.13)	7 (21.88)	15 (46.88)
	Total	0 (0.00)	18 (27.27)	24 (36.36)	24 (36.36)	18 (27.27)	42 (63.64)
Control *n* = 62	Second‐line chemotherapy (*n* = 32)	0 (0.00)	9 (28.13)	8 (25.00)	15 (46.88)	9 (28.13)	17 (53.13)
	Third‐line and beyond (*n* = 30)	0 (0.00)	7 (23.33)	8 (26.67)	15 (50.00)	7 (23.33)	15 (50.00)
	Total	0 (0.00)	16 (25.81)	16 (25.81)	30 (48.39)	16 (25.81)	32 (51.60)

In subgroup analysis, in the second‐line treatments, the ORR of control and experiment group were 28.13% and 32.35%, and DCR were 53.13% and 79.41%, respectively. There were significant differences in DCR between the two groups (*p* = 0.04). In the third‐line and beyond treatment, the ORR of control and experiment group were 23.33% and 21.88%, and DCR were 50.00% and 46.88%, respectively. The two groups showed no significant differences (*p* > 0.5) (Table 2).

### 3.3 Toxicity side effects

Toxicity was assessed for all 128 patients who received at least one dose of chemotherapy. Treatment‐related toxicities are summarized in Table 3. There was no statistical significance of toxicity side effects between two groups (*p* > 0.05). Most toxicities were grade 1 ~ 2. The incidence of grade 3–4 adverse events was low in both groups. The main adverse events in the experiment group were gastrointestinal reactions and hematological toxicity. Neutropenia and delayed diarrhea were characteristic adverse reactions of irinotecan. The experiment group showed similar incidence of grade 3–4 neutropenia and diarrhea as the control group (15.15% vs. 11.29%, *p* = 0.52; 10.61% vs. 9.68%, *p* = 0.86). All 128 patients were tolerated and no treatment‐related deaths. After G‐CSF, rehydration, and imodium therapy, most adverse reactions were alleviated.

**TABLE 3:** Toxicity side effects of two groups.

Toxicity	Grade1–2 [Cases (%)]			Grade 3–4 [Cases (%)]		
	Experiment	Control	*p*	Experiment	Control	*p*
Vomiting	12 (18.18)	11 (17.74)	0.95	6 (9.09)	5 (8.06)	0.84
Late diarrhea	25 (37.88)	23 (37.10)	0.93	7 (10.61)	6 (9.68)	0.86
Anemia	8 (12.12)	6 (9.68)	0.66	6 (9.09)	6 (9.68)	0.91
Neutropenia	28 (42.42)	25 (40.32)	0.81	10 (15.15)	7 (11.29)	0.52
Thrombocytopenia	6 (9.09)	5 (8.06)	0.84	2 (3.03)	2 (3.23)	0.95
Acute cholinergic syndrome	10 (15.15)	8 (12.90)	0.72	0 (0.00)	0 (0.00)	
Total				31 (46.97)	26 (41.94)	0.57

### 3.4 PFS and OS

The deadline of follow‐up was December 2021, and the median follow‐up time was 22.89.

Patient's condition progressed, we followed up with the patients on their subsequent treatment. Some patients underwent optimal supportive care, some received immune therapy combined with chemotherapy, while others underwent simple chemotherapy or immune therapy. There was no statistically significant difference in the subsequent treatment plans between the experimental group and the control group (Table 4).

**TABLE 4:** Posttreatment in two groups.

Posttreatment	Control (*n* = 62)	Experiment (*n* = 66)	*p* Value
Chemotherapy	11	13	0.98
Immunotherapy	15	16	
Chemotherapy+ immunotheray	8	7	
BSC	28	30	

**FIGURE. 2:**
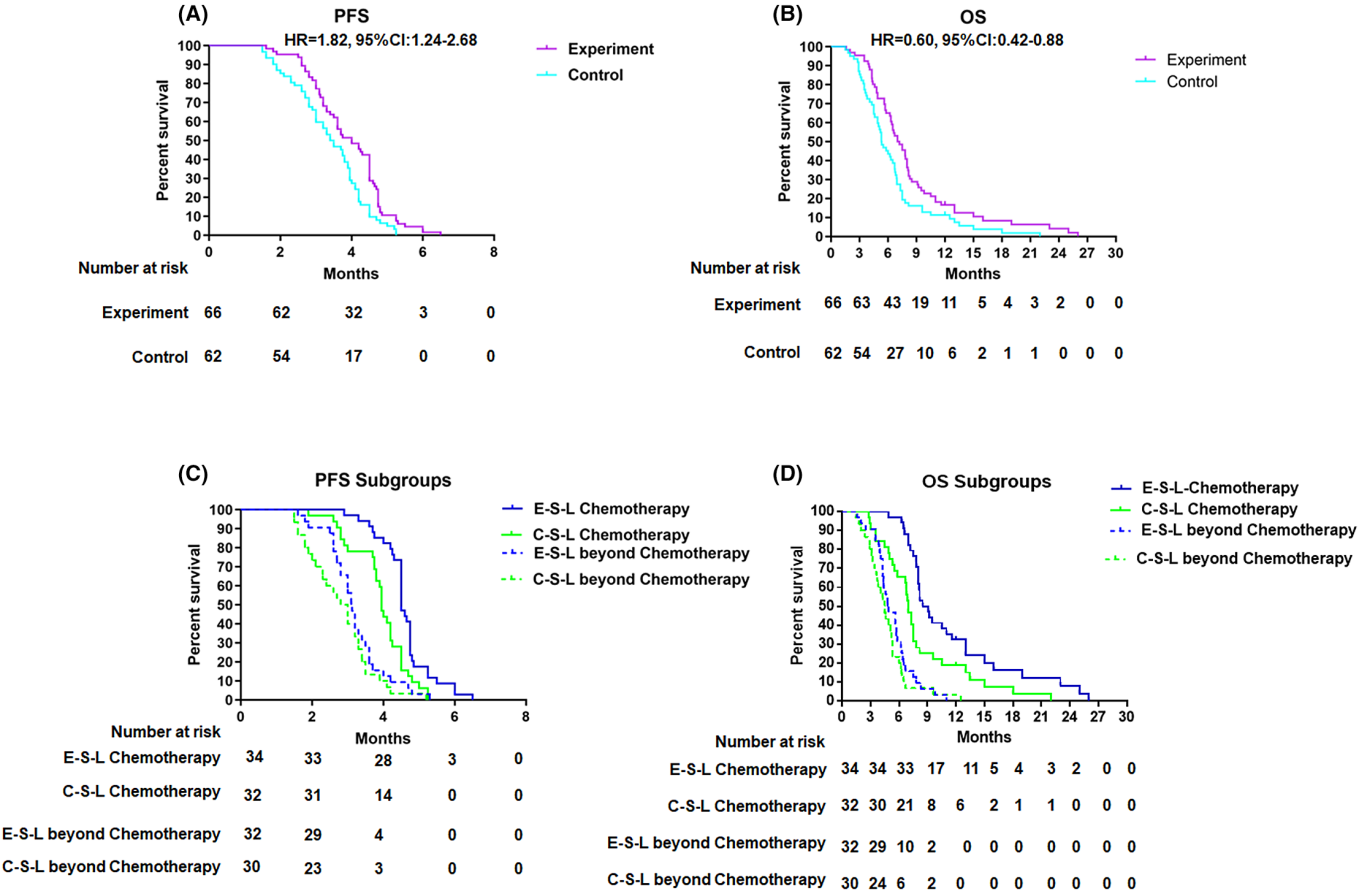
OS and PFS curves were estimated by the Kaplan–Meier method. (A) PFS of two groups. (B) OS of two groups. (C) PFS subgroup analysis for two groups. (D) OS subgroup analysis for two groups. C‐S‐L: control second line. E‐S‐L: experiment second line.

In the control group, the PFS and OS median of patients were 3.37 (95% CI: 3.00–3.90) months and 5.3 (95% CI: 4.43–6.88) months, respectively. In the experiment group, the PFS and OS median of patients were 3.91 (95% CI: 3.55–4.50) months and 7.0 (95% CI: 5.85–8.15) months, respectively. There was statistical significance of PFS and OS between two groups (PFS *p* = 0.002, OS *p* = 0.01) (Figure.2A,B).

In subgroup analysis in the second‐line treatment, the PFS median of control and experiment group were 3.90 (95% CI: 3.80–4.20) and 4.60 (95% CI: 4.50–4.75) months, respectively OS median were 6.95 (95% CI: 6.39–7.50) and 8.5 (95% CI: 7.24–9.76) months, respectively, which had statistically significant differences of PFS or OS between the two groups. (PFS *p* = 0.001, OS *p* = 0.005), in the third‐line and beyond treatment, the PFS medians of control and experiment group were 2.80 (95% CI: 2.30–3.25) months and 3.19 (95% CI: 2.85–3.35) months, OS median were 4.5 (95% CI: 3.83–5.17) months and 4.8 (95% CI: 3.69–5.91) months. But there was no significant difference of PFS or OS between the two groups (PFS *p* = 0.19, OS *p* = 0.31) (Figure 2C, D).

### 3.5 Effect of prior chemotherapy regimens on PFS and OS

**FIGURE. 3:**
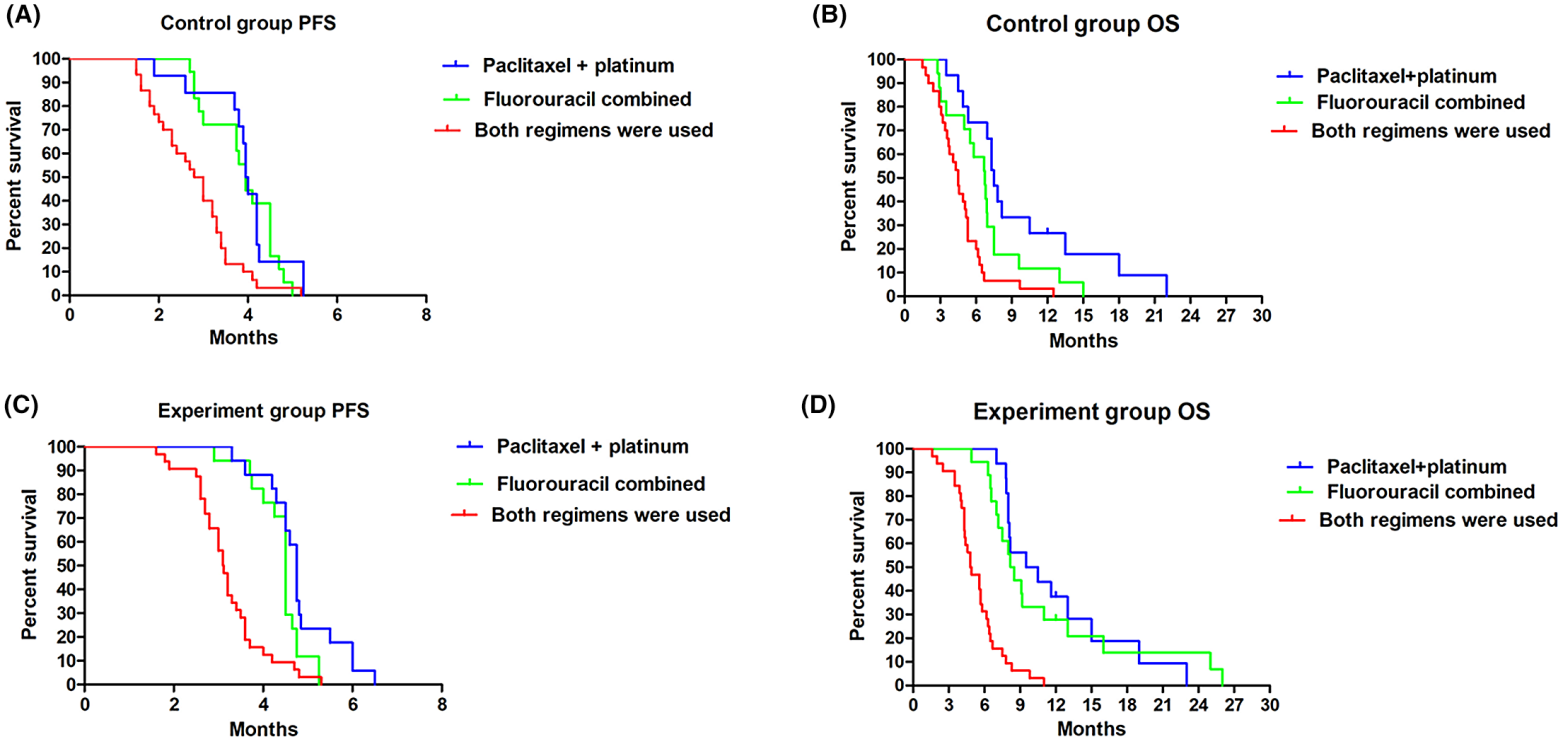
Effect of prior chemotherapy regimens on PFS and OS was estimated by the Kaplan–Meier method. (A) Effect of prior chemotherapy regimens on PFS in the control group. (B) Effect of prior chemotherapy regimens on OS in the control group. (C) Effect of prior chemotherapy regimens on PFS in the experiment group. (D) Effect of prior chemotherapy regimens on OS in the experiment group.

In the control group, the median PFS of fluorouracil combined therapy, paclitaxel + platinum therapy and both regimen therapy was 3.95 (95% CI: 2.90–4.50) vs. 3.98 (95% CI: 3.80–4.20) vs. 2.90 (95% CI: 2.20–3.40) months (Figure 3A), and median OS was 6.75 (95% CI: 5.41–8.10) vs. 7.5 (95% CI: 6.70–8.31), vs. 4.5 (95% CI: 3.83–5.17) months (Figure 3B), the median PFS or OS of fluorouracil combined therapy and paclitaxel + platinum therapy was higher than that of both regimen therapy (*p* < 0.05). In the experiment group, the median PFS of fluorouracil combined therapy, paclitaxel+platinum therapy and both regimen therapy was 4.50 (95% CI: 4.25–4.65) vs. 4.75 (95% CI: 4.50–4.85) vs. 3.11 (95% CI: 3.00–4.00) months (Figure 3C), and median OS was 8.0 (95% CI: 6.75–9.25) vs. 10.5 (95% CI: 6.38–14.62) vs. 4.8 (95% CI: 3.69–5.91) months (Figure 3D), the median PFS or OS of fluorouracil combined therapy and paclitaxel+platinum therapy was higher than that of both regimen therapy (*p* < 0.05). The results indicated fluorouracil combined therapy and paclitaxel+platinum therapy had no effect on the subsequent PFS and OS. However, both regimen therapy reduced the subsequent PFS and OS.

### 3.6 Effect of ECOG PS on PFS and OS

**FIGURE. 4:**
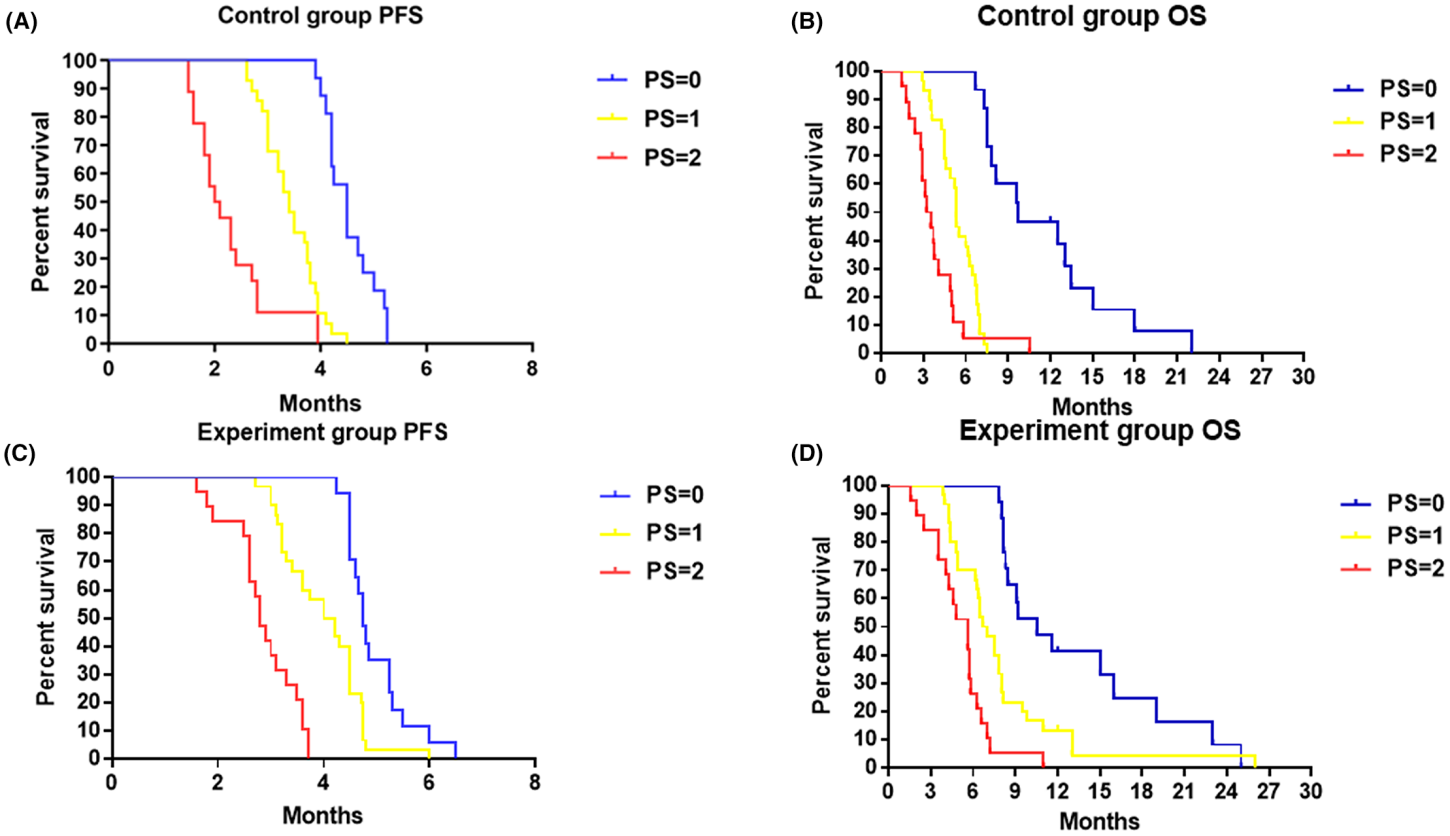
Effect of PS on PFS and OS was estimated by the Kaplan–Meier method. (A) Effect of PS on PFS in the control group. (B) Effect of PS on OS in the control group. (C) Effect of PS on PFS in the experiment group. (D) Effect of PS on OS in the experiment group.

In the control group, the median PFS of PS = 0, 1, and 2 was 4.50 (95% CI: 4.20–4.85) vs. 3.40 (95% CI: 3.00–3.95) vs. 2.05 (95% CI: 1.80–2.70) months (*p* < 0.05) (Figure 4A), and median OS was 9.70 (95% CI: 4.90–14.50) vs. 5.30 (95% CI: 5.10–5.50) vs. 3.20 (95% CI: 2.37–4.03) months (*p* < 0.05) (Figure 4B). In the experiment group, the median PFS of PS = 0, 1, and 2 was 4.75 (95% CI:4.50–5.25) vs. 4.10 (95% CI: 4.20–4.75) vs. 2.80 (95% CI: 2.60–3.30) months (*P* < 0.05) (Figure 4C), and median OS was 10.50 (95% CI: 7.14–13.86) vs. 6.7 (95% CI: 5.71–7.68) vs. 5.60 (95% CI: 4.19–7.01) months (*p* < 0.05) (Figure 4D). The results indicated that the higher the PS score, the worse the prognosis.

## 4 DISCUSSION

Esophageal carcinoma is a highly fatal malignancy in China, 95% of which are ESCC. Most patients are in advanced or metastatic stage at the time of diagnosis, and the prognosis is poor. ESCC is a different solid tumor from esophageal and esophagogastric junction adenocarcinoma with respect to biological and geographic distribution despite sharing the same anatomical location.^9^ Most international esophageal carcinoma guidelines recommend similar chemotherapy regimens for both ESCC and adenocarcinoma, while these recommendations are often derived from studies on both gastric and esophageal adenocarcinoma or only esophagogastric junction adenocarcinoma.^20^ Platinums, fluoropyrimidines, and taxanes were the main drug of first‐line chemotherapy for advanced and metastatic esophageal carcinoma.

Until now, apart from immune checkpoint inhibitors, there is currently no universally accepted alternative treatment regimen for advanced ESCC.^21^ But response rate of immunotherapy for PD‐L1 positive patients in the second‐line and beyond therapy is 18% ~ 28%, PFS only is 1.5 ~ 2.6 months, and OS is 6.8 ~ 10.8 months.^22^ What's more is it increased economic burden on most patients in China. Therefore, rescue chemotherapy is still important.

Irinotecan, a topoisomerase I inhibitor, has shown antitumor activity in the treatment of gastric, lung, cervical, and colorectal cancers. As irinotecan monotherapy has low ORR of 10%–15% in the treatment of cisplatin refractory esophageal carcinoma, more and more studies have explored irinotecan‐based regimens to get better clinical efficacy recently.^7^

In a retrospective study by Wang et al., a group of 27 advanced ESCC patients were treated with a combination of irinotecan and fluorouracil‐based regimens after the initial failure of platinum‐based treatment. The median PFS and OS for the patients were 4.8 and 10.5 months, respectively.^23^ Assersohn et al. demonstrated that a second‐line chemotherapy with irinotecan and 5‐Fu in esophagogastric carcinoma had 6.4 months median OS.^24^, ^25^ Williamson et al. carried out a phase II trial of gemcitabine + irinotecan in patients with esophageal cancer in 2006 and found the six‐month OS rate to be 56%.^26^ Huang et al. conducted a prospective phase 3 trial in 15 centers across China; they found that patients treated with irinotecan plus S‐1 had a significantly longer PFS and higher ORR than those treated with S‐1 monotherapy.^27^

Raltitrexed is a new generation anti‐metabolic cytotoxic drug that is similar to 5‐Fu, S‐1 or capecitabine. It was developed and marketed in the United Kingdom in 1996, while approved for use in China in 2010. A water‐soluble antifolate drug, based on quinazolone, is transported effectively into cells by the reduced folate carrier (RFC). The drug undergoes a significant polyglutamate process through the folylpolyglutamate synthase once inside the cells. Polyglutamation makes the drug over 100 times more active in inhibition and extends its retention time within cells.^28^ Raltitrexed (Tomudex) is a specific and long‐lasting inhibitor of TS, and plays a critical role in DNA synthesis and repair, and has been found to be involved in tumorigenesis or progression by several clinical studies. It has been widely used in chemotherapy for colorectal cancer, head and neck cancer, hepatic cancer, gastric cancer, cervical carcinoma, and malignant mesothelioma.^29^

Recently, raltitrexed has been applied to treat patients with esophageal carcinoma. A prospective study conducted by Sun et al. discovered that concurrent chemoradiotherapy based on raltitrexed/cisplatin was a successful treatment method for ESCC that is locally advanced.^18^ In the raltitrexed group, the PFS median was 29.1 months, and 1‐year PFS rate was estimated to be 80.4%. From 2015 to 2017, Qiu et al. launched a study of raltitrexed + nedaplatin combined with concurrent radiotherapy for advanced ESCC; they found the ORR was 90%, the 1 year and 2 year OS rate was 70.4% and 55.7%, respectively.^12^ These studies showed that raltitrexed was effective for ESCC. In addition, raltitrexed currently only pays about RMB 1000.00 for one treatment cycle for one patient in China, while irinotecan is fully reimbursed by the National Medical Insurance. Therefore, the irinotecan + raltitrexed treatment does not increase the financial burden of patients. However, the safety and efficacy of irinotecan combined with raltitrexed in the rescue treatment of advanced ESCC is still unknown.

Our study aimed to assess the effectiveness and secureness of using irinotecan and raltitrexed as second‐line and subsequent treatments for ESCC patients in the advanced stage. We found no difference in ORR between the two groups, but the subgroup analysis found that in the second‐line treatment group, the DCR of the experimental group was better than that of the control group. We found that PFS and OS of irinotecan combined with raltitrexed treatment was better than that of irinotecan monotherapy, there was statistical significance between two groups. In the stratified analysis, PFS and OS of the two groups were different (median PFS 3.9 m vs. 4.6 m, OS 6.95 m vs. 8.5 m) in the second‐line treatment, but there was no significant difference in the third‐line and beyond treatment. The results of our research showed fluorouracil combined therapy and paclitaxel+platinum therapy had no effect on the subsequent PFS and OS. However, both regimen therapy which was used reduced the subsequent PFS and OS. It indicated that these two regimens did not produce secondary resistance to irinotecan or raltitrexed, and did not affect subsequent use. It also indicated that fluorouracil and raltitrexed, although both belong to antimetabolites, did not produce cross‐resistance. However, if both treatment options were used, PFS and OS will be much worse indicating that it is easier to develop drug resistance after multiline chemotherapy, which affects the efficacy.

Dr Chen^13^ had carried out a retrospective study of irinotecan combined with raltitrexed in the treatment of advanced ESCC. He enrolled 38 patients, the median PFS was 105 days, the median OS was 221 days, which was similar to that of our study. He found that there were no differences in ORR, PFS, or OS between second‐line treatment and third‐line and beyond treatment. While PFS or OS between second‐line treatment was different in our study. This difference in results may be due to the different sample sizes of the two studies. This study also found that the higher the PS score and the shorter the PFS or OS, whether in the experimental or control group, indicating that the worse the general state, the poorer the body's tolerance to chemotherapy, and worse the prognosis, which was similar to previous studies.^30^ The incidence of side effects was similar in the two groups, the patients could tolerate it, and raltitrexed did not increase side effects.

## 5 CONCLUSION

In summary, our prospective study demonstrated the efficacy of irinotecan combined with raltitrexed for salvage chemotherapy of advanced ESCC prior to irinotecan monotherapy, and there is no increase in side effects. The PFS and OS of irinotecan + raltitrexed may be better than that of irinotecan monotherapy, especially in second‐line treatment, which should be confirmed with a phase III study including much more patients.

## AUTHOR CONTRIBUTIONS

**Xichao Dai:** Conceptualization (equal); writing – original draft (equal); writing – review and editing (equal). **Leilei Tao:** Investigation (equal); methodology (equal). **Jinqiu Wang:** Formal analysis (equal); investigation (equal); methodology (equal). **Wenjuan Wu:** Conceptualization (equal); writing – original draft (equal); writing – review and editing (equal). **Weigang Bian:** Investigation (equal); methodology (equal). **Xichun Dai:** Formal analysis (equal); investigation (equal); methodology (equal). **Surong Chen:** Formal analysis (equal); investigation (equal); methodology (equal).

## FUNDING INFORMATION

Grants from the National Natural Science Foundation of China (81802999) and Yancheng Medical Science and Technology Development Project (YK2018017) provided support for the study.

## CONFLICT OF INTEREST STATEMENT

The authors declare that they have no competing interests.

## ETHICS STATEMENT

The study was approved by the Institutional Review Board of First people's Hospital of Yancheng (IRB2020K016) and the Institutional Review Board of Northern Jiangsu People's Hospital (KY2020001).

## INFORMED CONSENT

All the participants signed an informed consent before participating in the study.

## Data Availability

The corresponding author can provide the datasets used or analyzed in the current study upon reasonable request.

## References

[1] Jemal A , Bray F , Center MM , Ferlay J , Ward E , Forman D . Global cancer statistics. CA Cancer J Clin. 2011;61(2):69‐90. 21296855 10.3322/caac.20107

[2] Siegel R , Ma J , Zou Z , Jemal A . Cancer statistics, 2014. CA Cancer J Clin. 2014;64(1):9‐29. 24399786 10.3322/caac.21208

[3] Lozano R , Naghavi M , Foreman K , et al. Global and regional mortality from 235 causes of death for 20 age groups in 1990 and 2010: a systematic analysis for the global burden of disease study 2010. Lancet (London, England). 2012;380(9859):2095‐2128. 23245604 10.1016/S0140-6736(12)61728-0 PMC10790329

[4] Crosby T , Evans M , Gillies RS , Maynard ND . The management of a patient with an operable carcinoma of the oesophagus. Ann R Coll Surg Engl. 2009;91(5):366‐370. 19622256 10.1308/003588409X432428 PMC2758428

[5] Huang FL , Yu SJ . Esophageal cancer: risk factors, genetic association, and treatment. Asian J Surg. 2018;41(3):210‐215. 27986415 10.1016/j.asjsur.2016.10.005

[6] Ohashi S , Miyamoto S , Kikuchi O , Goto T , Amanuma Y , Muto M . Recent advances from basic and clinical studies of esophageal squamous cell carcinoma. Gastroenterology. 2015;149(7):1700‐1715. 26376349 10.1053/j.gastro.2015.08.054

[7] Kim M , Keam B , Kim TM , et al. Phase II study of Irinotecan and cisplatin combination chemotherapy in metastatic, Unresectable Esophageal Cancer. Cancer Res Treat. 2017;49(2):416‐422. 27488873 10.4143/crt.2016.121 PMC5398400

[8] Domper Arnal MJ , Ferrandez Arenas A , Lanas AA . Esophageal cancer: risk factors, screening and endoscopic treatment in Western and eastern countries. World J Gastroenterol. 2015;21(26):7933‐7943. 26185366 10.3748/wjg.v21.i26.7933 PMC4499337

[9] Huang J , Xu J , Chen Y , et al. Camrelizumab versus investigator's choice of chemotherapy as second‐line therapy for advanced or metastatic oesophageal squamous cell carcinoma (ESCORT): a multicentre, randomised, open‐label, phase 3 study. Lancet Oncol. 2020;21(6):832‐842. 32416073 10.1016/S1470-2045(20)30110-8

[10] Tsunoda A , Yasuda N , Nakao K , et al. Phase II study of S‐1 combined with irinotecan (CPT‐11) in patients with advanced colorectal cancer. Oncology. 2009;77(3–4):192‐196. 19729976 10.1159/000236017

[11] Carnaghi C , Rimassa L , Garassino I , et al. Irinotecan and raltitrexed: an active combination in advanced colorectal cancer. Ann Oncol. 2002;13(9):1424‐1429. 12196368 10.1093/annonc/mdf229

[12] Qiu X , Li J , Zhou H , et al. Concurrent chemoradiotherapy with raltitrexed and nedaplatin regimen for esophageal squamous cell carcinoma. Medicine (Baltimore). 2020;99(4):e18732. 31977864 10.1097/MD.0000000000018732 PMC7004679

[13] Liu M , Jia Q , Wang X , et al. Clinical efficacy of irinotecan plus raltitrexed chemotherapy in refractory esophageal squamous cell cancer. Anticancer Drugs. 2020;31:403‐410. 31917701 10.1097/CAD.0000000000000891 PMC7077961

[14] Ghiringhelli F , Vincent J , Bengrine L , et al. Hepatic arterial chemotherapy with raltitrexed and oxaliplatin versus standard chemotherapy in unresectable liver metastases from colorectal cancer after conventional chemotherapy failure (HEARTO): a randomized phase‐II study. J Cancer Res Clin Oncol. 2019;145(9):2357‐2363. 31273511 10.1007/s00432-019-02970-8 PMC11810189

[15] Zhao P , Ding Z , Tang L , Zhou X . Preliminary investigation of intraperitoneal raltitrexed in patients with gastric cancer. World J Surg Oncol. 2014;12:403. 25547003 10.1186/1477-7819-12-403 PMC4396911

[16] Li XY , Liu L , Xie XM , Zhou C . The role of raltitrexed/cisplatin with concurrent radiation therapy in treating advanced cervical cancer. Eur Rev Med Pharmacol Sci. 2014;18(22):3491‐3496. 25491626

[17] Gaafar P , Sallam YA , Shafik HE , Aboulkassem FA , Emara M , Khaled H . Impact of new chemotherapeutic agents on the outcome of Egyptian patients with advanced malignant pleural mesothelioma. Gulf J Oncolog. 2014;1(16):56‐63. 25316393

[18] Ding WX , Liu S , Ma JX , et al. Raltitrexed increases radiation sensitivity of esophageal squamous carcinoma cells. Cancer Cell Int. 2019;19:36. 30820189 10.1186/s12935-019-0752-y PMC6378748

[19] Schwartz LH , Litière S , de Vries E , et al. RECIST 1.1‐update and clarification: from the RECIST committee. Eur J Cancer. 2016;62:132‐137. 27189322 10.1016/j.ejca.2016.03.081 PMC5737828

[20] Harada K , Rogers JE , Iwatsuki M , Yamashita K , Baba H , Ajani JA . Recent advances in treating oesophageal cancer. F1000Res. 2020;9:9. 33042518 10.12688/f1000research.22926.1 PMC7531047

[21] Sardaro A , Ferrari C , Carbonara R , Altini C , Lavelli V , Rubini G . Synergism between immunotherapy and radiotherapy in esophageal cancer: an overview of current knowledge and future perspectives. Cancer Biother Radiopharm. 2021;36(2):123‐132. 32551915 10.1089/cbr.2020.3643

[22] Baba Y , Nomoto D , Okadome K , et al. Tumor immune microenvironment and immune checkpoint inhibitors in esophageal squamous cell carcinoma. Cancer Sci. 2020;111(9):3132‐3141. 32579769 10.1111/cas.14541 PMC7469863

[23] Wang X , Wang X , Huang J . Irinotecan plus fluorouracil‐based regimen as second or third‐line chemotherapy for recurrent or metastatic esophageal squamous cell carcinoma. Thorac Cancer. 2016;7(2):246‐250. 27042229 10.1111/1759-7714.12323 PMC4773301

[24] Leary A , Assersohn L , Cunningham D , et al. A phase II trial evaluating capecitabine and irinotecan as second line treatment in patients with oesophago‐gastric cancer who have progressed on, or within 3 months of platinum‐based chemotherapy. Cancer Chemother Pharmacol. 2009;64(3):455‐462. 19104814 10.1007/s00280-008-0893-5

[25] Assersohn L , Brown G , Cunningham D , et al. Phase II study of irinotecan and 5‐fluorouracil/leucovorin in patients with primary refractory or relapsed advanced oesophageal and gastric carcinoma. Ann Oncol. 2004;15(1):64‐69. 14679122 10.1093/annonc/mdh007

[26] Williamson SK , McCoy SA , Gandara DR , et al. Phase II trial of gemcitabine plus irinotecan in patients with esophageal cancer: a southwest oncology group (SWOG) trial. Am J Clin Oncol. 2006;29(2):116‐122. 16601427 10.1097/01.coc.0000199883.10685.2b

[27] Huang J , Xu B , Liu Y , et al. Irinotecan plus S‐1 versus S‐1 in patients with previously treated recurrent or metastatic esophageal cancer (ESWN 01): a prospective randomized, multicenter, open‐labeled phase 3 trial. Cancer Commun (Lond). 2019;39(1):16. 30940189 10.1186/s40880-019-0359-7 PMC6444575

[28] Wei N , Zhang B , Wang Y , et al. Transarterial chemoembolization with raltitrexed‐based or floxuridine‐based chemotherapy for unresectable colorectal cancer liver metastasis. Clin Transl Oncol. 2019;21(4):443‐450. 30306400 10.1007/s12094-018-1942-0

[29] Kelly C , Bhuva N , Harrison M , Buckley A , Saunders M . Use of raltitrexed as an alternative to 5‐fluorouracil and capecitabine in cancer patients with cardiac history. Eur J Cancer. 2013;49(10):2303‐2310. 23583220 10.1016/j.ejca.2013.03.004

[30] Ghazy HF , El‐Hadaad HA , Wahba HA , Abbas R , Abbas OA . Metastatic esophageal carcinoma: prognostic factors and survival. J Gastrointest Cancer. 2021;53:446‐450. 33847917 10.1007/s12029-021-00610-4

